# BiopLib and BiopTools—a C programming library and toolset for manipulating protein structure

**DOI:** 10.1093/bioinformatics/btv482

**Published:** 2015-08-30

**Authors:** Craig T. Porter, Andrew C.R. Martin

**Affiliations:** Institute of Structural and Molecular Biology, Division of Biosciences, University College London, Darwin Building, Gower Street, London WC1E 6BT, UK

## Abstract

**Summary:** We describe BiopLib, a mature C programming library for manipulating protein structure, and BiopTools, a set of command-line tools which exploit BiopLib. The library also provides a small number of functions for handling protein sequence and general purpose programming and mathematics. BiopLib transparently handles PDBML (XML) format and standard PDB files. BiopTools provides facilities ranging from renumbering atoms and residues to calculation of solvent accessibility.

**Availability and implementation:** BiopLib and BiopTools are implemented in standard ANSI C. The core of the BiopLib library is a reliable PDB parser that handles alternate occupancies and deals with compressed PDB files and PDBML files automatically. The library is designed to be as flexible as possible, allowing users to handle PDB data as a simple list of atoms, or in a structured form using chains, residues and atoms. Many of the BiopTools command-line tools act as filters, taking a PDB (or PDBML) file as input and producing a PDB (or PDBML) file as output. All code is open source and documented using Doxygen. It is provided under the GNU Public Licence and is available from the authors’ web site or from GitHub.

**Contact:** andrew@bioinf.org.uk

## 1 Introduction

Handling PDB files is central to almost all research in structural bioinformatics and structural biology. Various reusable programming libraries have been developed for bioinformatics, but most libraries available for scripting languages focus on sequence. Both BioPerl (www.bioperl.org) and BioPython (www.biopython.org) provide modules for handling PDB structures which implement a PDB parser and data structure, but provide almost no routines for manipulating or analysing the data. A BioPython Google Summer of Code in 2010 added some more PDB handling code (biopython.org/wiki/GSOC2010_Joao), but this is not in the main distribution version. BioRuby (www.bioruby.org) provides a data structure for PDB data and a number of functions for finding records, but little for analysis.

BioJava (www.biojava.org) has a structure module, which handles input and output of standard PDB and mmCIF files, structural alignment using CE and FATCAT algorithms and access to CATH and SCOP. A number of C++ libraries are available and well-maintained including Protein Library (PL, protlib.uchicago.edu, part of the Open Protein Simulator, a protein folding simulator), Victor (Hirsh *et* *al.*, 2015), OpenStructure (Biasini *et* *al.*, 2013) and BALL (Hildebrandt *et* *al.*, 2010). The latter two also provide Python bindings. While C++ is favoured by some, C remains one of the most popular languages for computationally demanding tasks. Many major projects—both within computational biology (e.g. EMBOSS (Rice *et* *al.*, 2000) and our own ProFit, www.bioinf.org.uk/software/profit) and in general (e.g. the Linux kernel)—are implemented in C. EMBOSS again focuses on sequence, but with the STRUCTURE add-on package provides a small number of tools for manipulating protein structure. However, these are limited in scope and flexibility. Other Bioinformatics libraries are available, but are generally more specialized; we have provided a more comprehensive list at www.bioinf.org.uk/bioplib/libraries/.

Here, we introduce BiopLib, a C library for the manipulation of protein structure and sequence, and BiopTools, a set of programs written in C which use many of the functions implemented in the library. Development of BiopLib began in the late 1980s, and it has been enhanced over the last 25 years. Recently, we have made a concerted effort to standardize and document the code, extending it to handle PDBML format files and ensuring it will handle multi-character chain names. A recent announcement by the PDB stated that the largest PDB files (containing >62 chains or 99 999 ATOM records) would use multi-character chain names and will only be distributed in mmCIF and PDBML format (www.rcsb.org/pdb/static.do?p=general_information/whats_new.jsp?b=1412).

To our knowledge, BiopLib is the only C library for manipulation of protein structure which is regularly kept up to date and comes with a comprehensive set of tools for protein structure manipulation. Certainly, it is the only C library which supports PDBML as well as the standard PDB format in a transparent manner and, as a result, we believe that BiopTools provides the first solvent accessibility program able to handle PDBML files.

Previously, a number of programs included in BiopTools have been distributed on an *ad hoc* basis, but only the relevant BiopLib routines have been distributed with these programs, with the restriction that they may not be used for any other purpose without first obtaining a signed licence from us.

## 2 Implementation

All code in BiopLib and BiopTools is written in standard ANSI C with the exception that a compile-time flag can be set to make use of pipes, a standard POSIX.2 extension. This allows the PDB reading code to access a program to unpack compressed PDB files on-the-fly. Support for PDBML files is also controlled by a compile-time flag and exploits the libxml2 library for reading XML files (xmlsoft.org). All code compiles cleanly with full warnings turned on under the GCC C compiler to ensure portability of the code and avoid potential cross-platform bugs. While Linux is the primary development target, the code has also been tested under Windows (using MinGW) and Apple Mac environments. The code is maintained on GitHub.

### 2.1 BiopLib

The BiopLib library currently consists of ∼55 700 lines of C code including comments or ∼35 300 lines excluding comments. Most people working with PDB files only need access to a reliable set of coordinates. Generally, where a file contains multiple models and alternative occupancies, a program (e.g. to calculate solvent accessibility or to fit structures) requires a single set of coordinates and none of the other information that comes with the coordinates. This is the default behaviour of the BiopLib PDB reader. However, alternative occupancies or models can be read, and parsers are provided for key header records; CONECT and MASTER records are correctly handled if atom numbering changes. The library is in active development with ∼11 900 lines (∼7 500 without comments) added in the last 3 months and support for mmCIF format planned.

A key design philosophy has been to allow flexibility, rather than forcing the user into a particular way of working. This contrasts with existing C++ and other object-orientated libraries, which enforce a particular object model. As stated in the BioPython documentation for their structure module: *‘A macromolecular structure is represented using a structure, model, chain, residue, atom (or SMCRA) hierarchy.* *[…]* *Such a data structure is not necessarily best suited for the representation of the macromolecular content of a structure.’* In contrast, BiopLib is designed around the concept of a ‘PDB linked list’ which contains all the atoms for a given model and occupancy in the PDB file. Routines are provided to create a hierarchical data structure on top of this to allow the user to work with residues and chains, or to index the linked list to allow it to be accessed as an array. Thus, the user can select the approach that is best suited to the work in hand. Experiments have shown that the approach of allocating a structure for each atom gives no time penalty compared with allocating memory in larger chunks. On a slow laptop, reading 43 335 atoms and performing some calculations on the data took 1.113s using a linked list, 1.157s using a pre-allocated array.

The source code is extensively documented using Doxygen and an addition to Doxygen has been written to group functions in a logical fashion, so that related functions can easily be browsed. The Doxygen documentation for all BiopLib functions can be seen at www.bioinf.org.uk/software/bioplib/doc/html, but some of the key functions include: Read and write PDB files including handling PDBML files and multiple occupancies; Manipulate and modify the list of PDB coordinates; Calculate solvent accessibility; Find residues and atoms; Calculate distances, angles, torsions, centre of geometry and RMSD; fit structures and Align sequences. Mathematics functions include Eigen values and vectors and the shortest distance from a point to a line, while general programming functions include an interactive command parser and FORTRAN-style rigid-column file input.

### 2.2 BiopTools

BiopTools consists of ∼17 600 lines of C code (∼11 400 lines with comments removed), a relatively small amount since most of the complexity is contained within BiopLib.

A full list of tools in BiopTools is provided on the website, but some of the most significant programs include: extract the sequence from a PDB file; select specified atom types; extract a specified chain; extract a specified range of residues; add or remove hydrogens; renumbering; rotating and translating; solvent accessibility calculation and calculate backbone torsion angles.

Since many of the tools take PDB (or PDBML) as input and generate PDB (or PDBML) as output, and standard input/output are used if files are not specified, they can be chained together using Unix-style pipes. For example, to extract the C*α* atoms from chain L of compressed PDB file pdb1yqv.ent.gz, renumber the atoms and residues and save the result to the file out.pdb:

  pdbgetchain    L    pdb1yqv.ent.gz
    |    pdbatomsel    CA    |    pdbrenum    >    out.pdb

## 3 Availability

The software is released under the GNU Public Licence. A commercial licence is also available on request for those wishing to incorporate code into closed source applications.

Information on BiopLib, including extensive documentation, is available at www.bioinf.org.uk/software/bioplib. BiopTools is available at www.bioinf.org.uk/software/bioptools.
